# Isolation and Characterization of an Endophytic Fungus *Colletotrichum coccodes* Producing Tyrosol From *Houttuynia cordata* Thunb. Using ITS2 RNA Secondary Structure and Molecular Docking Study

**DOI:** 10.3389/fbioe.2021.650247

**Published:** 2021-06-17

**Authors:** Rajreepa Talukdar, Srichandan Padhi, Amit K. Rai, Marco Masi, Antonio Evidente, Dhruva Kumar Jha, Alessio Cimmino, Kumananda Tayung

**Affiliations:** ^1^Mycology and Plant Pathology Laboratory, Department of Botany, Gauhati University, Guwahati, India; ^2^Institute of Bioresources and Sustainable Development, Regional Centre, Gangtok, India; ^3^Department of Chemical Sciences, University of Naples Federico II, Naples, Italy

**Keywords:** *Houttuynia cordata*, *Colletotrichum coccodes*, antimicrobial activity, tyrosol, ITS2 RNA secondary structure

## Abstract

An endophytic fungus isolated from healthy leaf tissues of *Houttuynia cordata* Thunb., an ethnomedicinal plant of North East India, showed a considerable amount of antimicrobial activity. The ethyl acetate extract of the fungal culture filtrates displayed promising antimicrobial activity against a panel of clinically significant pathogens including *Candida albicans, Staphylococcus aureus*, *Escherichia coli*, and *Pseudomonas aeruginosa*. Bioassay guided purification of the organic extract using column and thin layer chromatography yielded a pure homogenous compound which was identified using spectroscopic methods (essentially by ^1^H NMR and MS) as tyrosol, a well-known phenylethanoid present in several natural sources. Besides, molecular docking studies against tyrosyl tRNA synthetases (TyrRS) of *S. aureus* (PDB ID: 1JIL) and *E. coli* (PDB ID: 1VBM), and CYP45014α-lanosterol demethylase (CYP51) of *C. albicans* (PDB ID: 5FSA) revealed tyrosol has a strong binding affinity with the enzyme active site residues. The fungus was identified as *Colletotrichum* sp. and characterized by its genomic ITS rDNA and ITS2 sequences. Phylogenetic analyses showed clustering of our isolate with *Colletotrichum coccodes.* Species of *Colletotrichum* are also reported to be plant pathogens. Therefore, to confirm the endophytic lifestyle of the isolate, ITS2 RNA secondary structure study was undertaken. The result indicated our isolate exhibited differences in the folding pattern as well as in motif structures when compared to those of pathogenic *C*. *coccodes*. The findings indicated that endophytic fungi harboring *H. cordata* could be explored as a potent source of antimicrobial agents.

## Introduction

Exploring the secondary metabolites produced by microorganisms colonizing unusual or unique environmental settings have turned on new possibilities toward discovering novel compounds of therapeutic interest and developing them as lead drugs. Endophytic fungi associated with the plants used in the traditional medicines are of special interest as these live in between the intercellular spaces of healthy tissues and cause hardly any infection while also producing a plethora of novel chemical entities with diverse pharmacological and biotechnological applications (Strobel and Daisy, 2003). Furthermore, the symbiotic association of these endophytes with the host plants indicates their compounds to be less toxic to the cell and does not kill the eukaryotic host system (Shubin et al., 2014; Chutulo and Chalannavar, 2018). The total number of fungal species including the endophytes is estimated to be 1.5 million, of which only 6–7% has been described till now, while the remaining still waiting to be introduced to the existing world of microbes. Besides, it is also estimated that 51% of bioactive substances isolated from these endophytic fungi were previously unknown (Hawksworth, 2001; Kharwar et al., 2011). On the other hand, bioinformatics approaches such as molecular docking have emerged as a usual and essential component of drug discovery and design pipeline. It assigns and employs a variety of conformation search strategies to the receptor and ligands, and predicts possible binding affinity by establishing chemical interactions between them (Ruyck et al., 2016; Padhi et al., 2020).

Northeast India, a major part of the Indo-Burma Belt, is recognized as a global biodiversity hotspot owing to its unique habitat and rich diversity of endemic flora and fauna. This region is a treasure-house of a large number of medicinal plants which are being used by various ethnic tribes for the treatment of various ailments. However, a repertoire of them remains untapped despite their tremendous potential in traditional medicines. Herein, an endophytic fungus identified as *Colletotrichum* sp. was isolated from the healthy leaf tissues of *Houttuynia cordata* Thunb., a potential ethnomedicinal plant being used for the treatment of stomach related disorders. The organic extract of its culture filtrates displayed considerable antimicrobial activity against some clinically significant human test pathogens. The isolate was also characterized in terms of ITS rDNA sequence and ITS2 secondary structures. The bioactive extract which was purified by chromatographic and spectroscopic techniques yielded a pure compound identified as tyrosol. Molecular docking studies revealed that the compound possesses a high binding affinity toward specific bacterial and fungal enzymes responsible for their growth and pathogenesis.

## Metabolites and Methods

### Isolation of the Source Organism

The fungus was isolated from healthy leaf tissues of *H. cordata* collected from Sonapur (26° 06′ 57.5″ N 91° 58′ 44.8″ E), Assam, India predominantly inhabited by tribal communities. A voucher specimen of the plant species has been deposited at the herbaria of Department of Botany, Gauhati University with accession number 18538 upon identification. Briefly, for isolation of endophytic fungi, healthy leaves were washed thoroughly with tap water followed by washing with 10% mild bio-detergent and finally with double distilled water to remove the surface debris. Surface sterilization of the leaves was carried out following standard protocol with slight modifications (Jena and Tayung, 2013). The leaves were dipped sequentially in 70% ethanol (3 min), followed by 0.5% sodium hypochlorite (NaOCl) (2 min), and then rinsed thoroughly with sterile distilled water (1 min). Finally, the leaves were dried over sterile blotting paper inside the laminar airflow chamber. Surface sterilized leaves measuring about 0.5 mm in diameter were then punched out using a sterile puncture and were inoculated onto Petri plates containing potato dextrose agar (PDA) medium supplemented with streptomycin sulfate (50 μg/ml). The plates with the leaf fragments were then incubated at 25 ± 2°C for 2 weeks and observed once a day for the growth of mycelia. Hyphal tips growing out of the leaf fragments were transferred to PDA slants, sub-cultured, and stored at 4°C. The isolates were identified based on their morphological and microscopic characters referring to standard identification manuals (Gilman, 1971; Barnett and Hunter, 1996; Damm et al., 2012). The fungus used in the present study was one of the several endophytic fungal isolates obtained from the surface-sterilized leaf fragments which showed considerable antimicrobial activity during the preliminary antimicrobial assay.

### Metabolites Extraction and Determination of Antimicrobial Activity

The fungus was cultivated in potato dextrose broth (PDB) and incubated at 28°C in BOD shaking incubator for 2 weeks at 120 rpm. After the specified incubation period, the culture broth was filtered through sterile Whatman filter paper to remove the mycelia mat. The filtrate so obtained was collected and fungal metabolites were extracted using an equal volume of ethyl acetate (EtOAc) through vigorous shaking for 10–15 min in a separating funnel. The solvent was then evaporated in a rotary evaporator and the organic extract thus obtained was weighed. The antimicrobial activity of the extracts was determined by Agar cup diffusion assay against some clinically significant test pathogens. The test organisms included three bacterial pathogens, namely, *Staphylococcus aureus* (MTCC 737), *Pseudomonas aeruginosa* (MTCC 424), and *Escherichia coli* (MTCC 443), and one pathogenic fungus, *Candida albicans* (MTCC 227). The test organisms were procured from the Microbial Type Culture Collection (MTCC), Institute of Microbial Technology (CSIR-IMTECH), Chandigarh, India. The test organisms were activated by cultivating them on freshly prepared nutrient agar (NA) and sabouraud dextrose agar (SDA) media, respectively for bacterial and fungal pathogens. Meanwhile, sterile NA and SDA plates were prepared and the plates were inoculated with 0.2 ml of overnight grown bacterial and fungal cultures containing 1.0 × 10^6^ cells. A lawn culture was prepared on each plate by evenly spreading the inoculums with the help of a sterile cotton swab. Agar cups were prepared in the plates by scooping out the solid medium with a sterile cork borer (7 mm in diameter). The cups were then filled in with 100 μl of the EtOAc organic extracts dissolved in 10% dimethyl sulfoxide (DMSO) at a concentration of 1 mg/ml and incubated respectively at 37 ± 1°C for 24 h for bacterial and at 28°C ± 1°C for 48 h for fungal pathogens. The assay was carried out in triplicates and antimicrobial activity was determined by the appearance of clear zones of inhibition against the test organism around the agar cups. 10% DMSO was used as the negative control for the assay.

### Minimum Inhibitory Concentration

The minimum inhibitory concentration (MIC) of the organic extract was determined by micro-broth dilution assay in a sterile 96-well plate (Padhi and Tayung, 2013) against *P. aeruginosa*, *S. aureus*, *E. coli*, and *C. albicans.* A two-fold dilution of the extracts with the concentration ranging from 1,000 to 62.5 μg/ml was made. The wells were filled with 90 μl of each test bacterial and fungal suspension (approx. 10^6^ CFU/ml). Test extracts (10 μl) of different concentrations were added into each well to make upto a final volume of 100 μl. Medium containing 10% DMSO was used as the negative control. After incubation at 37°C for 24 h for bacteria and 48 h for fungal pathogens, a solution (10 μl) of triphenyltetrazolium chloride (TTC) was added to each well as microbial growth indicator, and the microplates were incubated for an additional 30 min. MIC was determined as the lowest concentration of the test extract at which no pink color appeared.

### Purification and Characterization

The organic extract obtained from *Colletotrichum coccodes* HCS3 culture filtrates (99.9 mg) was purified on a silica gel (0.063−0.200 mm, Kieselgel 60, Merck) column eluted with chloroform and isopropanol (CHCl_3_-*i*-PrOH 9:1) and a specific volume of eluent (5 ml) was collected in vials. The fraction contained in each vial was subjected to thin-layer chromatography (TLC). The plates were visualized by exposure to UV radiation or spraying first with 10% sulfuric acid (H_2_SO_4_) in methanol (MeOH) and then with 5% phosphomolybdic acid in ethanol (EtOH), followed by heating at 110°C for 10 min on a hot plate. The vials containing related fractions were clubbed into one vial affording seven homogeneous fractions. The fractions were concentrated in a rotary evaporator, weighed, and evaluated for antimicrobial activity at 1 mg/ml against the test organisms using an agar well diffusion assay. The active fractions were further purified by analytical and preparative TLC eluted with CHCl_3_-*i*-PrOH (9:1) yielding a pure and homogenous compound. To elucidate the structure of this pure compound and also to check the purity, ^1^H NMR spectra were recorded in deuterated chloroform (CDCl_3_) at 500 MHz on a Varian instrument. The same solvent (CDCl_3_) was used as an internal standard. Electrospray Ionization/Mass Spectrometry(ESI/MS) and liquid chromatography (LC)/MS analyses were performed using the LC/MS time-of- light (TOF) system (Agilent 6230B, HPLC 1260 Infinity) column Phenomenex LUNA [C18 (2), 5 μm, 150 mm × 4.6 mm]. Analytical and preparative TLC was carried out on silica gel (0.25 and 0.5 mm, respectively, F254, Kieselgel 60, Merck) and reversed-phase (0.20 mm, F254, Kieselgel 60 RP-18, Merck) plates.

### Molecular Docking

Based on the antimicrobial activity of the EtOAc extract, molecular docking was carried out to examine the possible binding affinity of the isolated compound toward TyrRS of *S. aureus* (1JIL) and *E. coli* (1VBM), and CYP51 of *C. albicans* (5FSA). The 3D crystal structures of these enzymes were retrieved from Protein Data Bank (PDB) and optimized (removal of unwanted water molecules, heteroatoms, and addition of polar hydrogens, missing amino acids) using the “prepare protein” protocol of Discovery Studio (DS) Client v20.1.0.19.295. Similarly, the 2D structure of the metabolite was optimized using the “prepare ligand” protocol. The active site of the enzymes was selected as the binding site for the ligand and a docking experiment was performed using the DS CDOCKER program which is an implementation of a CHARMm based docking tool (Gagnon et al., 2014). Docking optimization was carried out with a root mean square threshold (RMSD) 0.5 Å and pose cluster radius 0.5 to ensure the docked poses are diverse. The pose with the highest negative interaction energy was selected as the best binding conformation. The entire experiment was carried out following the procedure mentioned by Padhi et al. (2021).

### Genomic DNA Isolation, Amplification, and Sequencing

Species confirmation of the endophytic isolate was determined by ITS rDNA sequence analysis. The fungal strain was cultured on PDB and a small amount of the mycelia was suspended in 40 μl MQ water. Genomic DNA was isolated by the CTAB method (Clarke, 2009). A portion of the genomic DNA was diluted upto 50 ng/μl for use in PCR. The nuclear ribosomal DNA and ITS region of the isolate were amplified using the universal primers ITS5 (5′-GGAAGTAAAAGTCGTAACAAGG-3′) and ITS4 (5′-TCCTCCGCTTATTGATATGC-3′). The PCR was set up using the following components: 2.5 μl buffer (109), 1.5 μl MgCl_2_ (25 mM), 2.5 μl dNTPs (2 mM), 0.2 μl Promega Taq (5 U/μl), 1.0 μl each of forward and reverse primers (5 pm/μl) and 6.0 μl DNA from the diluted extract. The PCR condition was run with an initial denaturation at 94°C for 3 min. Denaturation, annealing, and extension were done at 96°C for 10 s, 55°C for 10 s, and 72°C for 30 s, respectively, in 45 cycles. The final extension was done at 72°C for 10 min and held at 4°C. After the PCR cycle, 2 μl of the product was used to check on 1% agarose gel. DNA sequencing was performed using an ABI 3730 sequencer. The forward and reverse sequence reads thus obtained were assembled to generate the contig using CAP3, a bioinformatics tool used for the assembling of genomic DNA reads (Huang and Madan, 1999). The annotated ITS rDNA contig was submitted to GenBank and an accession number was obtained.

### Identification of Microorganisms

A homology search and analysis for the ITS rDNA sequence as obtained above was performed with the Basic Local Alignment Search Tool (BLAST). Based on the results, 995 ITS rDNA sequences belonging to *Colletotrichum* spp. and having similarity with the isolate were randomly selected and retrieved from GenBank. The sequences were further filter searched and altogether 83 sequences with complete ITS rDNA and 30 sequences with ITS2 which were trimmed from the ITS rDNA using ITS2 secondary structure database (Koetschan et al., 2010) were selected for phylogenetic study. For phylogenetic analysis, multiple sequence alignments were performed using CLUSTALW software utilizing default settings, and trees were generated by the character state Maximum Parsimony (MP) method using MEGA 6.0 (Tamura et al., 2013). The robustness of the tree was assessed by bootstrap analysis with 1,000 replications. Based on the phylogenetic analysis, selected ITS2 sequences belonging to *C. coccodes* of varied lifestyles (endophytic and pathogenic) were used to generate the RNA secondary structure using the mfold web server (Zuker, 2003). The structure prediction was performed with a temperature of 37°C; ionic conditions: 1 M NaCl, no divalent ions; the maximum number of nucleotides in a bulge or loop: 30; maximum asymmetry of an interior/bulge loop: 30; percentage sub-optimality number: 5 and upper bound on the number of computed foldings: 50. The structure chosen from different output files was the one with the highest negative free energy if various similar structures were obtained. The consensus ITS2 secondary structures were compared among different lifestyles and differences in their folding patterns as well as in number and types of motifs were investigated.

## Results

### Isolation and Identification of the Endophytic Fungus

**FIGURE 1:**
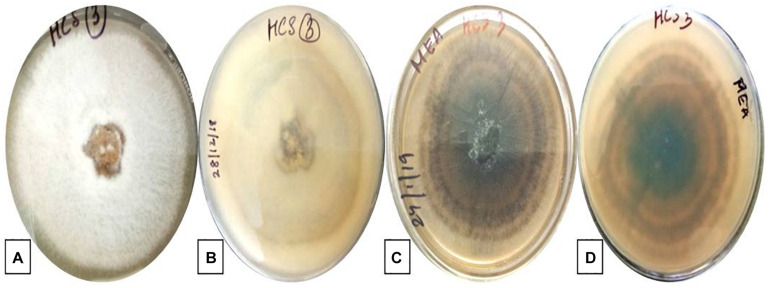
Colony morphology of *Colletotrichum coccodes*. (HCS3) on PDA: **(A)** front view, **(B)** reverse view and on MEA: **(C)** front view, **(D)** reverse view.

**FIGURE 2:**
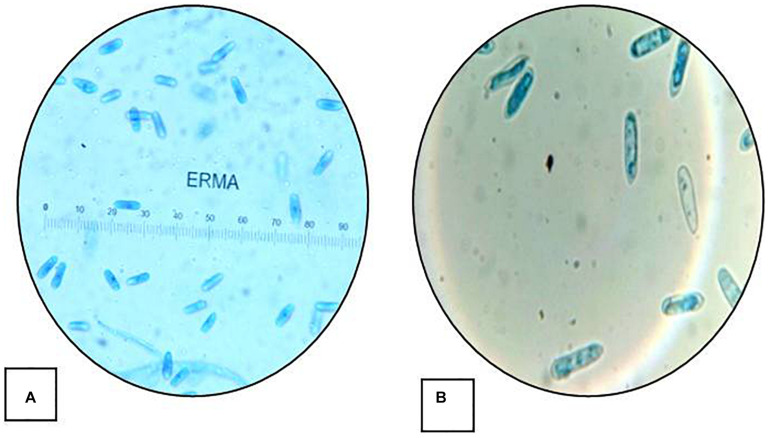
Conidial morphology *Colletotrichum coccodes*. (HCS3): **(A)** conidia (20–22 μm × 5–7 μm) (40×) and **(B)** conidia (100×).

The isolate described in this study was designated as strain HCS3 and was one among the several isolates that showed antimicrobial activity against the test pathogens used. The isolate was identified as *Colletotrichum* sp. based on the colony morphology and microscopic reproductive structures. The isolate was grown on PDA and malt extract agar (MEA) for identification. Colonies on PDA were circular, white initially with an orange tint that turns gray with age and darker at the center with black spots. Aerial mycelia white to grayish, cottony without visible conidial masses. The reverse side was white to pale orange in color. Growth on PDA was slow showing a diameter of about 55 mm in 7 days at 25°C (Figures 1A,B). While colonies on MEA were white with an orange tint at first but turned gray to dark blackish later. Colonies showed concentric ring-like growth both on front and reverse sides. Aerial mycelia were dark gray, sparse, and turned darker with age with conidial masses. The growth rate on MEA was faster showing a diameter of 67 mm in 7 days at 25°C (Figures 1C,D). Conidia measures 20–22 × 5–7 μm and is aseptate, smooth-walled, hyaline, and cylindrical with rounded ends, often with conspicuous papillate basal scars, guttulate, and slightly constricted centrally (Figure 2).

### Antimicrobial Activity and Determination of MIC

**FIGURE 3:**
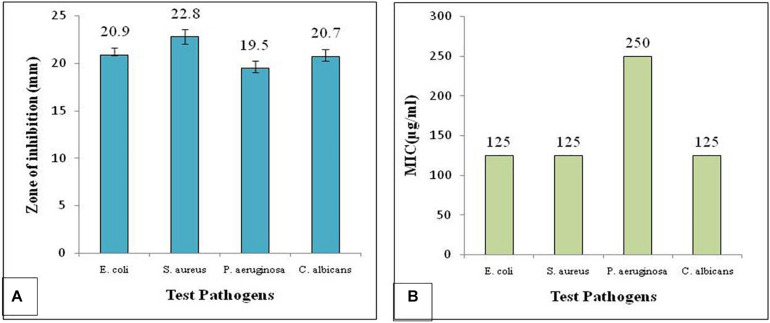
Antimicrobial activity of the *Colletotrichum coccodes* (EtOAc extract) showing **(A)** zone of inhibition and **(B)** MIC against test pathogens.

The results showed the organic extract exhibited considerable antimicrobial activity against all the test pathogens used. However, maximum inhibition was observed against *S. aureus* followed by *E. coli* and *C. albicans* (Figure 3A). MIC of the organic extract was determined by micro-broth dilution assay in sterile 96-well plate against all the test pathogens, *P. aeruginosa*, *S. aureus*, *E. coli*, and *C. albicans.* Different concentration of the organic extract such as 1,000, 500, 250, 125, and 62.5 μg/ml was used to determine the MIC. The wells were filled with 90 μl of each test bacterial and fungal suspension with 10 μl of different concentrations of the test extract. The MIC for *S. aureus*, *E. coli* and *C. albicans* was found to be 125 μg/ml and that for *P. aeruginosa* was 250 μg/ml (Figure 3B).

### Purification and Characterization of Tyrosol

**FIGURE 4:**
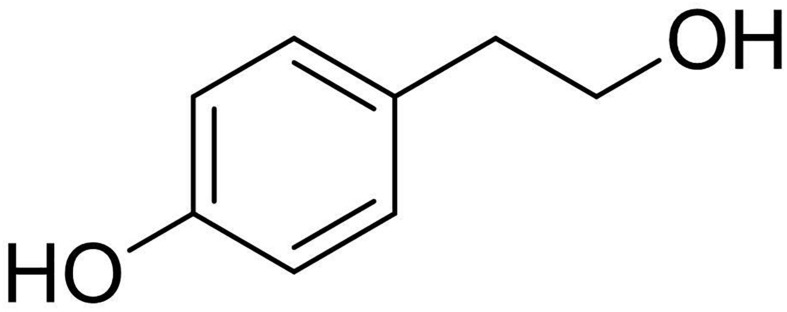
Chemical structure of tyrosol isolated from *Colletotrichum coccodes* bioactive extract.

The EtOAc extract was purified on column chromatography and yielded 7 homogenous fractions namely F1 (3.5 mg), F2 (4.0 mg), F3 (7.6 mg), F4 (2.6 mg), F5 (1.8 mg), F6 (1.5 mg), F7 (2.1 mg), and MeOH washes (37.4 mg). Among all, the fraction F3 was observed to be having substantial activity against the test pathogens, and the residue was further purified on TLC to yield a pure and homogenous solid (2.3 mg). ^1^H NMR spectrum of this compound showed signal system characteristics for a *p*-substituted benzene derivative and a hydroxyethyl residue. The compound was identified as “tyrosol” (Figure 4) by comparing its spectroscopic properties with those available in the literature. The identification was further validated and confirmed by ESI-MS spectrum recorded in a positive modality which showed the dimer sodiated [2M + Na]^+^, and sodiated [M + Na]^+^ adduct ions at *m/z*: 299 and 161, respectively. The spectroscopic descriptions of tyrosol are given below;

Tyrosol: ^1^H NMR (500 MHz), δ: 7.10 (d, *J* = 8.2 Hz, H-2 and H-6), 6.79 (d, *J* = 8.2 Hz, H-3 and H-5), 3.82 (t, *J* = 6.6 Hz, H_2_-8), 2.80 (t, *J* = 6.6 Hz, H_2_-7); ESIMS (+), *m/z*: 299 [2M + Na]^+^, 161 [M + Na]^+^.

### Molecular Docking

Molecular docking was carried out to understand the binding interaction and affinity of tyrosol toward the enzymes TyrRS of *S. aureus* and *E. coli*, and CYP51 of *C. albicans*. The isolated compound showed varied affinity toward the active sites of different enzymes used. The affinity was highest against TyrRS of *S. aureus* (CDOCKER energy −26.8457 kcal/mol; interaction energy −30.7030 kcal/mol) followed by that of *E. coli* (CDOCKER energy −24.8513 kcal/mol; interaction energy −29.3093 kcal/mol). The binding affinity of the tyrosol was also observed to be substantial against CYP51 of *C. albicans*. The binding between tyrosol and TyrRS of *S. aureus* was represented by two covalent C–H and three non-covalent (2 H-bond and 1 Pi-Alkyl) interactions. Similarly, the interaction between tyrosol and TyrRS of *E. coli* was characterized by 1 covalent C–H and 6 non-covalent ones including 1 Pi-alkyl and 5 H-bonds. However, the test ligand tyrosol was found to be bonded to the *C. albicans* CYP51 active site with the support of five non-covalent bonds including 3 H-bond, 1 Pi-Alkyl, and 1 Pi-Pi T shaped bonds. The CDOCKER energy and interaction energy for this binding was respectively computed to be −20.7595 and −24.2725 kcal/mol. The types of interaction, bond distance, and amino acids involved between tyrosol and the target enzymes are given in Tables 1, 2.

**TABLE 1:** Molecular docking of tyrosol into the active sites of TyrRS (*S. aureus* and *E. coli*) and CYP51 (*C. albicans*).

Description	Docking of ligand into enzyme active site	Enzyme–ligand interactions^∗^	Docking energies (kcal/mol)
*S. aureus* TyrRS-tyrosol docking	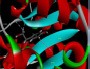	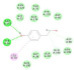	CDOCKER energy: −26.8457 Interaction energy: −30.703
*E. coli* TyrRS-tyrosol docking	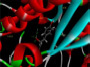	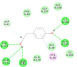	CDOCKER energy: −24.5813 Interaction energy: −29.3093
*C. albicans* CYP51-tyrosol docking	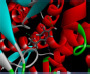	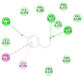	CDOCKER energy: −20.7595 Interaction energy: −24.2725

** 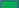 : Conventional hydrogen bonds,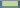 : Van der waals,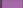 : Pi-Sigma or Pi-Stacked or Pi-Pi T Shaped and 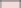 : Alkyl or Pi-Alkyl.*

**TABLE 2:** Receptor-ligand interactions between tyrosol and TyrRS (*S. aureus* and *E. coli*) and CYP51 (*C. albicans*).

Receptor–ligand interactions								
TyrRS (*S. aureus*)-tyrosol			TyrRS (*E. coli*)-tyrosol			CYP51 (*C. albicans*)-tyrosol		
ASR	IT	BD (Å)	ASR	IT	BD (Å)	ASR	IT	BD (Å)
TYR 36	H-bond	2.00	CYS 38	Pi-Alkyl	5.18	TYR118	H-bond	2.92
LEU 70	Pi-Alkyl	5.16	ASP 41	C-H bond	2.87	PHE 233	Pi-Pi T Shape	5.68
ASP 80	C-H bond	2.75	ASP 81	H-bond	2.06	LEU 376	Pi-Alkyl	4.65
ASP 177	H-bond	2.26	TYR 175	H-bond	1.96	HIS 377	H-bond	2.60
GLN 196	C-H bond	2.77	GLN 195	H-bond	2.01	MET508	H-bond	1.86
			ILE 196	H-bond	1.94			
			GLN 201	H-bond	2.79			

*ASR, active site residues, IT, interaction types; BD, bond distance; H-bond, conventional hydrogen bond; C–H bond, carbon–hydrogen bond.*

### Molecular Identification and Phylogenetic Analyses

**FIGURE 5:**
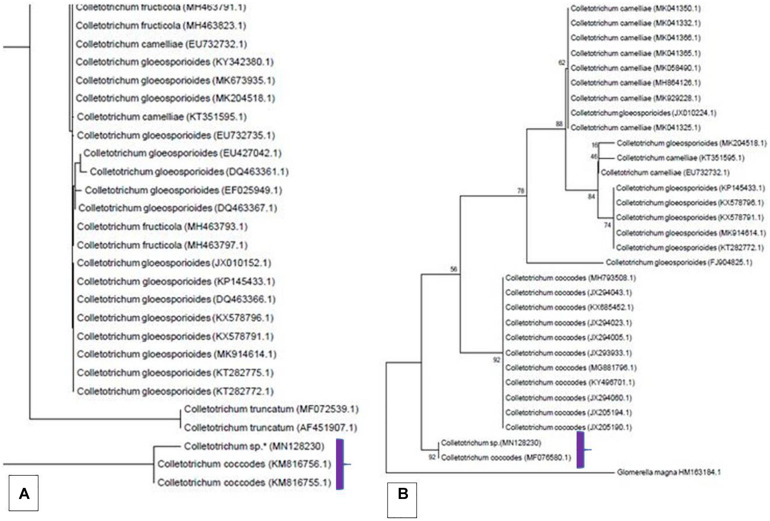
Snapshots of phylogenetic tree generated using Maximum Parsimony (MP) method showing clustering of our isolate under the clade *Colletotrichum coccodes*. Trees were constructed using ITS rDNA **(A)** and ITS2 **(B)** sequences.

The genomic DNA of the isolate was isolated and the region for ITS rDNA was amplified and sequenced using universal primers ITS4 and ITS5. The contig thus obtained from assembling the forward and reverse reads were deposited to the GenBank with the accession MN128230.1. A BLAST homology searches ITS rDNA was carried out against the NCBI non-redundant nucleotide (nr) database and the results showed our isolate resembling that of *Colletotrichum coccodes* (MF076580.1) with Maximum Identity 98.25%; Query Coverage 91% and E-value 0.0. Based on this, a total of 995 ITS rDNA sequences belonging to different *Colletotrichum* spp., were randomly retrieved from GenBank and screened for the presence of complete ITS rDNA region (18S rDNA-ITS1-5.8S-ITS2-rDNA-28S rDNA). Phylogenetic analysis was performed using selected 83 sequences and the tree showed clustering of our isolate under the clade *C. coccodes* (Figure 5A). Further, the sequences were trimmed for the ITS2, and as such a final tree was constructed using 30 ITS2 sequences. The MP phylogenetic tree displayed our isolate shared a close affinity toward *C. coccodes* which is supported by a bootstrap value 92 (Figure 5B).

### ITS2 Secondary Structure Analyses

**FIGURE 6:**
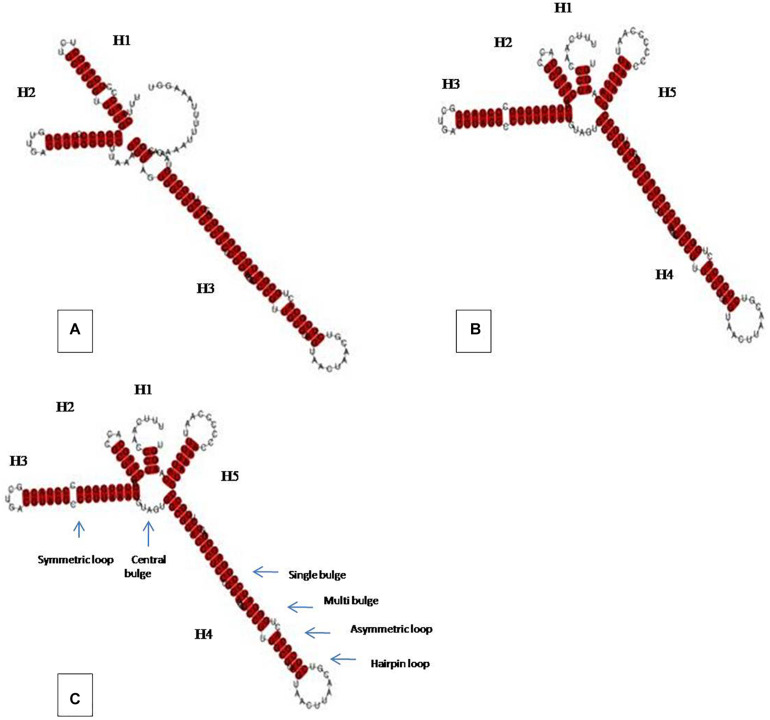
ITS2 secondary structures of *C. coccodes* among different life styles showing helices emerging from a central bulge, **(A)** pathogenic, **(B)** endophytic and **(C)** own isolate (MN128230.1). Motifs in the structures are indicated by arrow.

Prediction of secondary structures was carried out using selected 12 ITS2 sequences belonging to *C. coccodes* (11 pathogenic and 1 endophytic) and was compared to that of our isolate. Briefly, an ITS2 consensus structure for the pathogenic *C. coccodes* was generated and a comparative assessment between the pathogenic, endophytic lifestyles and our isolate was made. The consensus structure for pathogenic *C. coccodes* consisted of a conserved core bulge of which radiating three major helices (H1, H2, and H3), however, the ITS2 secondary structure for the endophytic *C. coccodes* as well as for that belong to our endophytic isolate comprised of one incomplete (H1) and four complete helices (H2, H3, H4, and H5) (Figure 6). The results displayed the pathogenic and the endophytic ITS2 structures exhibited different folding patterns and dissimilar structural motifs. Our isolate shared extreme similarity with that of endophytic lifestyle both in terms of ITS2 folding pattern as well as identical structural motifs. The details about different structural features among ITS2 structures of different lifestyles of *C. coccodes* are presented in Table 3.

**TABLE 3:** Comparison of motif features among *C. coccodes* of different life styles.

*C. coccodes* lifestyles	Hairpin loop (s)	Internal loop (s)		Bulges	
		Symmetric	Asymmetric	Single bulge	Multi bulge
Pathogenic	3	0	3	5	1
Endophytic	4	1	1	4	1
MN12830* (endophytic)	4	1	1	4	1

*Indicates own isolate.

## Discussion

Endophytic fungi which live in close association with vascular plants without causing any obvious symptoms are an important component of plant-micro ecosystems. They confer profound impacts on host plants as mutualists by promoting their growth and tolerance to diseases (Abdel-Azeem et al., 2019; Kaur, 2020). There is growing interest among researchers to isolate and characterize the endophytic fungi inhabiting medicinal plants as they have been producing a plethora of chemical entities of biotechnological and pharmaceutical significance. Besides, some of them have also the ability to produce the same bioactive metabolites as their host plants (Tan and Zou, 2001; Schulz et al., 2002; Gouda et al., 2016). In the present study, endophytic fungi colonizing leaf tissues of *H. cordata* Thunb., an ethnomedicinal plant of North East India was investigated. Among several other endophytic isolates, a fungus identified as *Colletrotrichum* sp. (HCS3), showed considerable antimicrobial activity against some clinically important pathogenic bacteria and fungi. The organic extract exhibited strong inhibition against *S. aureus*, *E. coli*, *P. aeruginosa*, and *C. albicans* with MIC range 125–250 μg/ml. The occurrence of *Colletotrichum* spp. as an endophyte is not rare; it has been isolated from several medicinal plants with potent biological activity against several drug-resistant pathogens (Lu et al., 2000; Arivudainambi et al., 2011; Sharma et al., 2017; Kim and Shim, 2019).

Molecular tools including phylogenetic analyses that aid along with the traditional techniques have been proven to be successful for correct identification of fungi and have further revolutionized the fungal classification (Sarwar et al., 2019). Moreover, the ITS region which consists of a highly conserved 5.8S rRNA and variable regions ITS1 and, ITS2, is one of the widely used phylogenetic markers for fungal species identification (Tamura et al., 2004; Xu and Adamowicz, 2016). Further, the spacer regions such as ITS1 and ITS2 are reportedly useful in detecting and identifying fungal pathogens from clinical specimens and environmental samples. Also, recent reports revealed the sequence variability in ITS2 is more apposite for phylogenetic reconstruction and species differentiation in eukaryotes as well as in fungi (Iwen et al., 2002). The resolution of the phylogenetic and evolutionary prediction can be improved further if structural features are taken into account as RNA secondary structures include morphological information that is not found in the sequence (Marinho et al., 2011; Heeg and Wolf, 2015; Zhang et al., 2020). Undeniably, ITS2 secondary structure plays a critical part in rRNA processing and formation of functional 60S subunit. Despite considerable variation in its nucleotide sequences, a typical eukaryotic ITS2 secondary structure (also known as four-helix or ring-pin model) consists of four helices that are maintained through several common and shared structure motifs including internal loops, hairpin loops, and bulges (Schultz et al., 2005; Kapoor et al., 2018). These structure motifs impart stability to the ITS2 structure and are reportedly susceptible to polymorphisms that further give rise to non-canonical G–U pairs within the conserved regions of double-stranded helices (Cote and Peculis, 2001; Freire et al., 2012). Above all, closely related species share very similar folding patterns of ITS2 with nearly identical structure motifs (Rampersad, 2014; Verma et al., 2020). Homology search and phylogenetic analysis using ITS as well as ITS2 sequence of the isolate (HCS3) revealed it to be *Colletotrichum coccodes* as the trees displayed clustering of our isolate under *C. coccodes*. Furthermore, a comparison of ITS2 secondary structure features revealed close affinity among pathogenic and endophytic *C. coccodes* including significant differences in the folding pattern and motif structures. Similar observations have also been put forth by several studies (Padhi and Tayung, 2013; Padhi et al., 2016) which suggested ITS2 structure could be used as a phylogenetic marker to establish similarities and variations between different lifestyles of the same species.

The EtOAc-derived organic extract which showed promising activity in inhibiting the growth of test pathogens was further purified to yield a potent phenolic compound identified to be tyrosol based on its NMR and ESIMS spectra. Tyrosol is a well-known secondary metabolite produced by both plants (Capasso et al., 1992) and fungi including *Diplodia seriata* (Venkatasubbaiah and Chilton, 1990), *Alternaria tagetica* (Gamboa-Angulo et al., 2001), *Neofusicoccum parvum* (Evidente et al., 2010), and *Neofusicoccum australe* (Andolfi et al., 2012), *Lasiodiplodia* spp. (Cimmino et al., 2017), *Diaporthella cryptica* (Cimmino et al., 2018). Moreover, several studies have also reported tyrosol from the bioactive extracts of endophytic *Colletotrichum* (*Colletotrichum gleosporoides*, *Colletotrichum crassipes*) (Anisha and Radhakrishnan, 2017; Masi et al., 2018; Plotnikov and Plotnikova, 2020). However, the report of its isolation from *C. coccodes* is limited. Interestingly, in the present study tyrosol was isolated from an endophytic *C. coccodes* extract having significant antimicrobial activity. This suggests the potential of endophytic *C. coccodes* colonizing *H. cordata* as producers of bioactive metabolites.

Natural products are often considered for their rich gallows which let them acting as ligands for various clinically relevant receptors and enzymes. The position of enzyme inhibitors as candidate drugs is very cosmic because of their expediency in treating several ailments. Searching inhibitors of enzymes responsible for the growth and maintenance of microbes (for example, enzymes involved in DNA replication, protein translation, cell wall synthesis, etc.) have become a familiar intent for antimicrobial drug discovery (Pereira et al., 2017; Belete, 2019). Tyrosyl tRNA synthetases (TyrRS) belonging to enzyme class aminoacyl tRNA synthetase have been playing an essential role in the faithful translation and protein synthesis in the bacterial cell. Several studies on natural products have targeted and validated inhibition of this enzyme for novel and effective therapeutics development against clinically significant bacterial pathogens such as *E. coli, S. aureus*, and *Mycobacterium tuberculosis* (Xiao et al., 2011; Zhu et al., 2015; Skupinska et al., 2017; Sun et al., 2017). Similarly, “lanosterol-14α-demethylase” which is a cytochrome P450 enzyme, has been reported to be having supreme significance in the biosynthesis of sterols in eukaryotic cells. The enzyme catalyzes the conversion of lanosterol to ergosterol, an important component of the fungal cell membrane, depletion of which leads to growth inhibition or disruption of cells. Because of this fact this enzyme has long been considered as a target of interest for most antifungal drug discovery against *Aspergillus niger*, *Cryptococcus albidus*, and especially against *C. albicans* (Kelly et al., 1990; Hargrove et al., 2017; Stana et al., 2017; Rana et al., 2019). Furthermore, the determination of crystal structures of these enzymes has led to the evolution of computational approaches which have been extremely advantageous in screening a large number of small molecules against specific targets of interest. Molecular docking examines and evaluates the affinity of probable drug candidates toward the binding cavities of target proteins or enzymes by establishing chemical connections of various strengths (Alam and Khan, 2018; Padhi et al., 2020). Tyrosol has earlier been reported as an inhibitor of several enzymes including α-glucosidase, cytochrome oxidase through *in silico* and *in vitro* investigations (Zhang et al., 2019; Yadav et al., 2020). Amini et al. (2017) reported the possible antibacterial action of tyrosol as it inhibited ATPase activity in *E. coli*. Nevertheless, reports describing the antimicrobial action mechanism of tyrosol are truly limited. In this study, the purified compound tyrosol was screened against the active sites of TyrRS of *S. aureus* and *E. coli*, and CYP51 of *C. albicans* using CDOCKER docking program. It was observed that the compound made significant contacts with the active site amino acids of all the target enzymes. As revealed from the energy parameters, tyrosol has the highest affinity toward *S. aureus* TyrRs followed by that of *E. coli.* Similarly, the binding affinity of tyrosol was also found to be substantial with *C. albicans* CYP51. The non-covalent bonds formed during any receptor-ligand interactions use to participate in a major role in pharmaceutical drug designing and development. The H-bonds usually confer structural stability and rigidity to the protein-ligand complexes. Moreover, there exist Pi-Sigma interactions (Pi-anion, Pi-cation, and Pi-alkyl) and salt bridges that reportedly maximize the binding affinity between protein and ligands in a physiological environment (Rahman et al., 2016; Aljoundi et al., 2020). In the present study, the interaction between tyrosol and target enzymes (TyrRS and CYP51) witnessed some non-covalent forces inclusively H-bonds and Pi-Sigma. This proposes that the antimicrobial activity of the organic extract obtained from *C. coccodes* culture filtrates could be linked to the presence of tyrosol which in turn might hinder the normal growth of the test bacteria and fungus by inhibiting the activity of the target enzymes. The findings further suggest that endophytic *C. coccodes* inhabiting medicinal plants like *H. cordata* Thunb. could be exploited as a potential producer of antimicrobial agents. Moreover, the *in silico* findings can be used in combination with *in vitro* enzymatic assays to investigate the efficacy of tyrosol as a potent enzyme inhibitor and an effective antimicrobial agent.

## Conclusion

In this study, bioactive extract of an endophytic *C. coccodes* isolated from the leaves of a medicinal plant *H. cordata* Thunb. was characterized to yield a pure metabolite tyrosol. Molecular docking revealed tyrosol has a strong affinity toward bacterial tyrosyl tRNA synthetase and fungal CYP45014α-lanosterol demethylase, suggesting its possible antimicrobial action mechanism. The isolate was characterized by ITS rDNA and ITS2 sequence, and ITS2 structure was used to differentiate our isolate from the same species of different lifestyles. The findings of the study could be useful in exploring the bioactive potential of fungal endophytes colonizing plants of ethnomedicinal importance. Further, ITS2 could be used as a potential marker for species differentiation among dissimilar lifestyles. To the best of our knowledge, tyrosol was isolated from *C. coccodes* for the first time.

## Data Availability Statement

The datasets presented in the study can be found at NCBI GenBank with the following URL: https://www.ncbi.nlm.nih.gov/nuccore/MN128230.

## Author Contributions

RT, KT, and DJ designed, carried out the experimental works, and drafted the manuscript. SP and AR contributed to the molecular docking and phylogeny. AC, MM, and AE did purification and characterization of the metabolite. All authors contributed to the article and approved the submitted version.

## Conflict of Interest

The authors declare that the research was conducted in the absence of any commercial or financial relationships that could be construed as a potential conflict of interest.
